# Assessing key mental health-related lifestyle behaviours and experiences in a single scale: a validation of the *Personal Experiences in Everyday Life* (PEEL) questionnaire

**DOI:** 10.1186/s12887-026-06790-x

**Published:** 2026-03-30

**Authors:** P. Tang, K. Kostyrka-Allchorne, A. Murray, M. Stoilova, E. Azeri, S. Livingstone, E. Sonuga-Barke

**Affiliations:** 1 Department of Psychology, School of Biological and Behavioural Sciences, Queen Mary University of London, London, UK; 2 School of Psychology, University of Edinburgh, Edinburgh, UK; 3 Department of Media and Communications, London School of Economics and Political Science, London, UK; 4 Department of Child and Adolescent Psychiatry, Institute of Psychiatry, Psychology & Neuroscience, King’s College London, London, UK

**Keywords:** Adolescence, Anxiety, Depression, Lifestyle behaviours, Negative social experiences, Psychometrics, Wellbeing

## Abstract

**Background:**

Adolescence is a period of heightened vulnerability to mental health problems, with lifestyle habits and negative social experiences representing both risk and resilience factors. Existing tools typically assess a limited range of such habits and experiences. The Personal Experiences in Everyday Life (PEEL) questionnaire is a brief measure of key modifiable lifestyle behaviours and negative social experiences. We examined its psychometric properties and associations with symptoms of depression, anxiety, and psychological wellbeing.

**Methods:**

Two community samples, a mid-adolescent (*n* = 552; mean age = 13.7, SD = 0.5, range 13–14; 48% female) and a late-adolescent sample (*n* = 383; mean age = 19.0, SD = 1.7, range 16–25; 76% female), were recruited from secondary schools/universities and completed the PEEL, assessing positive (e.g., ‘Sport/exercise’) and risky (e.g., ‘Smoking/vaping) lifestyle behaviours, and negative social experiences (e.g., ‘Being bullied’). We examined the PEEL’s factor structure, internal consistency, test-retest reliability and the associations with mental health and wellbeing, including moderation by sex and developmental stage. The RCADS-25 and DASS-21 measured depressive and anxiety symptoms; scores were standardised (z-scores) and pooled.

**Results:**

A three-factor solution for the PEEL items was supported across both samples: *Positive Activities*, *Unhealthy Diet*, and *Risky Activities*. For conceptual clarity, Risky Activities were divided into *Substance Use* and *Negative Social Experiences*, yielding four internally consistent subscales with good test-retest reliability. All factors were significantly associated with depression, anxiety, and wellbeing; Negative Social Experiences showed the strongest associations with depression and anxiety. Stronger positive associations between Substance Use and anxiety were evident in females, and stronger negative associations between Substance Use and Negative Social Experiences with wellbeing were seen in males. Stronger associations between Negative Social Experiences and depression and anxiety occurred in younger adolescents. Unhealthy Diet was positively associated with wellbeing in younger adolescents but negatively associated in older adolescents.

**Conclusions:**

The PEEL is an efficient and valid measure of adolescents’ lifestyle behaviours and negative social experiences. Negative social experiences accounted for much of the observed associations with mental health, highlighting the role of social context alongside lifestyle behaviours. The PEEL may support research seeking to understand or modify lifestyle and social drivers of adolescent mental health.

**Supplementary Information:**

The online version contains supplementary material available at 10.1186/s12887-026-06790-x.

## Background

Adolescence marks a time of intense physical, psychological, and social changes that occur in parallel with key educational transitions and the formation of personal identity. For many adolescents, strong foundations are built for future health, occupational success, and wellbeing during this critical developmental stage, but this period can also mean heightened psychological vulnerability [1, 2]. For a substantial proportion of young people, adolescence coincides with increased stress, emerging mental health problems, and the onset of psychiatric disorders [3].

Recent findings from the 2019 Global Burden of Disease study highlight the scale of adolescent mental health problems and the burden they carry. Over 11% of individuals aged 5 to 24 years, that is, 293 million people globally, live with at least one diagnosable mental disorder. Moreover, mental disorders account for approximately 20% of all years lived with disability in this age group, and nearly 25% of the lifetime burden of mental illness occurs before age 25 [4]. Consequently, youth mental health has become a central concern in global public health. Together, these data underscore adolescence as a critical window for understanding and potentially modifying mental health risk [5].

Because depression, anxiety, and low wellbeing are common in adolescence and may negatively influence academic performance, social relationships, and later life outcomes [6, 7], it is essential to identify modifiable factors that can support psychological resilience and reduce mental health risk. Lifestyle behaviours offer promising targets. Habits such as regular physical activity, a balanced diet, and engagement in creative or social leisure are not only important for physical and cognitive development, but also have demonstrated links to mental health outcomes [8–11]. For instance, physical activity and practices like mindfulness and meditation in adolescence have been linked to improved outcomes, such as emotional regulation, stress management, and mental health [12, 13]. Non-physical leisure activities, such as reading, writing, and creative hobbies, offer adolescents opportunities for self-expression, mastery, and distraction, and provide an enjoyable way to pass the time that may promote psychological wellbeing [14–17].

Conversely, lifestyle risks, such as substance use or an unhealthy diet, are consistently linked to poorer mental health. These behaviours may affect mood, coping, and social functioning through multiple biological and psychosocial pathways [18, 19]. Substance use can be both a risk factor and an outcome of psychological difficulties [20, 21], while an unhealthy diet, particularly high consumption of energy-dense, processed foods and sugary drinks, has been associated with systemic inflammation, metabolic dysregulation and increased risk of mental health problems [19, 22].

Despite the clear relevance of these lifestyle behaviours, existing measurement approaches in adolescent research are often limited. Many studies examine only one or two behaviours, such as physical activity, diet, or sleep, in isolation, which restricts the ability to assess the cumulative effects of multiple concurrent behaviours and limits insight into how multiple lifestyle factors may interact in everyday life [23]. For example, whether the lack of positive hobbies exacerbates the risk for substance use, or whether regular physical activity can reduce the risk of an unhealthy diet. Moreover, lifestyle habit measures typically do not capture key negative personal and social experiences, such as bullying, teasing, or school-related conflict, which in themselves are important drivers of mental health and often co-occur with lifestyle risks. Prosocial experiences and activities (e.g. volunteering, spending time with friends) foster belonging, self-esteem, and purpose [24, 25], whereas negative social experiences are strongly linked to depression, anxiety, substance use, and self-harm [26, 27]. School-based experiences, such as truancy or conflict with teachers, may signal early psychological difficulties and, in turn, contribute to social alienation or academic disengagement [28, 29]. Taken together, these findings highlight the need for an assessment tool that situates lifestyle behaviours within their broader social and interpersonal context.

To address these limitations, we developed the Personal Experiences in Everyday Life (PEEL) questionnaire - a brief measure of youth daily lifestyle habits and key negative social experiences. The primary purpose of the PEEL is to provide a concise measure that can be easily integrated into large research batteries, offering a profile of lifestyle behaviours and social experience risks. As a single questionnaire, the PEEL provides several advantages over combining standalone instruments. First, it allows efficient and consistent assessment of multiple lifestyle behaviours in large health-related research batteries and reduces respondent burden. Second, it facilitates comparability and harmonisation across studies, supporting meta-analyses and so encourages broad adoption. Finally, it includes items assessing important negative social experiences which, although not lifestyle habits per se, provide important context for understanding youth mental health. Therefore, the PEEL deliberately integrates lifestyle habits and negative social exposures to provide a broader context for mental health, instead of functioning purely as a lifestyle scale.

The present study aims to validate the PEEL as a measure of positive and risky lifestyle behaviours (exercise, hobbies, substance use, diet) and key personal experiences (bullying, teasing, and school conflict) across mid- and late adolescence, with the upper age range also capturing some emerging adults (i.e., 13–25 years). It also examines how modifiable lifestyle behaviours and key personal experiences uniquely and jointly relate to symptoms of anxiety, depression and wellbeing, generating evidence for the PEEL’s predictive validity. Although the PEEL was originally developed for adolescents, it was applied across a broader age range to capture both late adolescence and early adulthood. We note that while some items (e.g., school-related experiences) are more relevant to mid-adolescence, the measure broadly reflects lifestyle behaviours and social experiences across this developmental period.

Sex and developmental stage are examined as moderators to test whether the psychometric performance and predictive validity of the PEEL subscales were consistent across subgroups. Numerous studies suggest that lifestyle behaviours and mental health differ by sex. For example, males may engage more frequently in lifestyle risk behaviours, while females often report higher rates of depressive and anxiety symptoms [30, 31]. Developmental stage is also important – the same behaviour may have different implications in mid- versus late adolescence, partly because of differences in autonomy, peer influence, or academic contexts. Therefore, including sex and developmental stage (mid- vs. late adolescence) as moderators allows us to test whether the predictive validity of the PEEL domains vary across subgroups and to identify potential differences that may inform future longitudinal or intervention development research.

Our study has five research questions:

1. What is the factor structure, internal consistency, and test-retest reliability of the PEEL questionnaire, measuring lifestyle behaviours and negative social experiences, in mid- and late adolescence?
2. How well does the PEEL demonstrate predictive validity, as indicated by the unique and combined contribution of positive (e.g. exercise, creative hobbies) and risky lifestyle behaviours (e.g. substance use, poor diet) to symptoms of depression and anxiety and wellbeing?
3. Do negative social experiences (e.g. bullying, teasing, school conflict) explain variation in depression and anxiety symptoms and wellbeing over and above lifestyle behaviours, and, therefore, provide evidence for incremental validity of this domain within the PEEL?
4. Does sex moderate the associations between PEEL domains and symptoms of depression, anxiety, and wellbeing?
5. Does the PEEL demonstrate evidence of developmental moderation, that is, do associations between lifestyle behaviours, negative social experiences, and mental-health and wellbeing outcomes differ between mid- and late adolescence?

Overall, the present study will psychometrically validate the PEEL and evaluate whether its domains function similarly across sex and developmental stage, providing evidence on how lifestyle behaviours and negative social experiences relate to mental health and wellbeing outcomes in males and females and across different developmental stages.

## Methods

### Sample

This study used data from two community-based adolescent samples. The first and mid-adolescence sample was recruited from UK secondary schools and included 552 Year 9 students aged between 13 and 14 years (mean = 13.7, SD = 0.5; 48% female, 48% male). These adolescents participated in a longitudinal study on mental health, and the current study used their baseline data only. The second and late adolescence sample consisted of 383 young people aged between 16 and 25 years (mean = 19.0, SD = 1.7; 76% female, 24% male), who were recruited by circulating information about the study in three UK secondary schools identified using the research team’s professional networks. The study advertisement was also shared via two universities’ research volunteers’ recruitment systems.

Both samples represented a diverse ethnicity background (55% White, 15% South/East Asian, 8% Black, 18% mixed/other ethnicities in sample one, and 49% White, 30% South/East Asian, 9% Black, and 11% mixed/other ethnicities in sample two; and a wide socioeconomic spectrum (23% and 17% being eligible for free school meals - indicating socioeconomic deprivation due to limited household financial resources, respectively). All participants received the information sheet in a digital format and provided written consent online; informed consent was additionally obtained from parents or legal guardians for participants under the age of 16.

### Study design and procedure

This is a cross-sectional observational study. For the mid-adolescent sample, researchers agreed on the data collection procedure with the participating schools. Information about the study was shared with adolescents and their parents. Adolescents completed the study online via Qualtrics (https://www.qualtrics.com) in a school-based session between January and April 2024 and received a £15 shopping voucher as a thank you. Participants in the late adolescence sample read about the study information and completed assessments via Qualtrics or Gorilla (https://www.gorilla.sc) between April 2023 and March 2024. A subset of the late adolescence sample completed the PEEL twice (*n* = 145), approximately ten days apart (mean = 11.5; median = 10; range = 4 to 38). All participants received a £10 shopping voucher or three course credits (for those recruited via universities), depending on their preference.

Ethical approvals were received from the London School of Economics and Political Science (reference 249287) for the mid-adolescence sample and the London School of Economics and Political Science (reference 18934) and Queen Mary University of London (reference PSY2023‐39A) for the late adolescence sample.

### Measures

*Lifestyle behaviours and negative social experiences.* Lifestyle factors and social experiences were measured with the newly developed Personal Experiences in Everyday Life (PEEL) questionnaire. It was designed to capture day-to-day habits and experiences that are most salient to mental health, with input from young people aged 12–17 years to make sure the items are relevant and relatable. The PEEL was developed as part of a larger project on online experiences and youth mental health [32, 33]. The PEEL contains 19 items concerning positive lifestyle behaviours (e.g., ‘Sport or exercise’, ‘Hobby or creative activities’), risky lifestyle behaviours (e.g., ‘Drinking alcohol’, ‘Eating sweets, crisps, cakes, or ready-to-eat meals’), and negative social experiences (e.g., ‘Being bullied’, ‘Getting put down because of my ethnicity’). Respondents rated how often they had done or experienced each item in the past two weeks on a 5-point scale, from 0 = never to 4 = at least every day. Following the exploratory and confirmatory factor analyses, subscale scores were calculated as the average of the corresponding items, with higher scores indicating more engagement or exposure to the respective activity or experience.

*Mental health and wellbeing.* Participants in the mid-adolescence sample and a subset of the late-adolescence sample (those recruited from the secondary schools; total *n* = 743) completed the self-reported Revised Child Anxiety and Depression Scale 25 – Youth Version (RCADS-25), a 25-item measure of the severity of depression and anxiety with excellent validity and reliability in school-based adolescents [34]. Depression and anxiety scores were calculated respectively from the 10-item depression subscale (e.g., ‘I feel sad or empty.’) and 15-item anxiety subscale (e.g., ‘I worry that something bad will happen to me.’). Respondents rated how often each statement applied to them on a 4-point scale (0 = never to 3 = always). Because the RCADS-25 is not validated for use in adults over 18 years, the remaining participants in the late-adolescence sample (*n* = 192) completed the Depression, Anxiety, and Stress Scale (DASS-21), a validated 21‐item questionnaire of depression, anxiety and stress [35]. Scores were calculated from the respective depression and anxiety subscales, consisting of 7 items each, answered from 0 = did not apply to me at all to 3 = applied to me very much or most of the time.

Wellbeing was measured by the Warwick-Edinburgh Mental Wellbeing Scale (WEMWBS) in both samples, a 14-item measure of psychological wellbeing (e.g., ‘I’ve been feeling confident’) [36]. Respondents rated each item on a scale from 0 = none of the time to 5 = all of the time. A total score was calculated, with a higher score indicating better mental wellbeing.

*Demographics*. Participants also provided information about their age, biological sex, ethnicity, and eligibility for free school meals, which was used as an indicator of socioeconomic disadvantage in the UK.

### Statistical analysis

First, we calculated the means and standard deviations (SD) of the PEEL items in both samples and presented the descriptive statistics.

Second, we examined the psychometric properties of the PEEL. Although the item ratings are ordinal (0–4), we treated them as continuous, which is commonly accepted for 5-point Likert scales with moderate-to-large samples [37]. To examine the factor structure of the PEEL, we conducted an initial exploratory factor analysis (EFA) with principal axis factoring and direct oblimin rotation, using data from the late-adolescence sample. Although the items could be treated as formative indicators of lifestyle habits instead of reflecting a single underlying latent factor, we aimed to identify clusters of behaviours and subscales that can be scored separately, rather than a single composite score. We chose this sample for the initial factor extraction because the frequency of the items, especially regarding substance use, was rated higher than in the younger adolescents. The number of extracted factors was determined based on the Minimum Average Partial (MAP) test, visual inspection of the scree plot, the proportion of variance explained by each factor, and the conceptual coherence of the factor solutions [38]. Items that had small loadings (< |0.40|) or loaded on more than one factor (i.e., difference in loadings between primary and secondary factors < |0.20|), and factors that only had one loading item were removed before re-running the factor analysis.

Next, we applied the same process to data from the mid-adolescent sample to compare the factor structure of the PEEL across developmental stages and select a set of items with good validity in both mid- and late adolescence. The second EFA conducted in the mid-adolescent sample was partly confirmatory to test whether the factor structure from the initial EFA could be replicated. Finally, to validate and confirm the factor structure, we adopted a split-half EFA-CFA approach using the combined mid- and late-adolescence samples. The combined sample was randomly split into two subsamples; an exploratory factor analysis was conducted in subsample 1, and the resulting structure was examined using confirmatory factor analysis in subsample 2. Model fit was evaluated using multiple fit indices, including the Comparative Fit Index (CFI), the Tucker-Lewis Index (TLI), and the root mean square error of approximation (RMSEA).

Internal consistency of the PEEL was examined by Cronbach’s alpha for each subscale. Test-retest reliability was calculated using the subset of the late adolescence sample who completed the PEEL twice, approximately one week apart. Sex (female vs. male) and developmental stage (late vs. mid-adolescence) differences in the resulting subscale scores were examined by independent t-tests.

Third, we transformed the depression and anxiety scores measured by the RCADS-25 and DASS-21 into standardised Z-scores within each measure, respectively, before pooling the Z-scores together as the indicators of depression and anxiety symptoms across the whole sample. This approach was considered acceptable given the strong conceptual overlap between the two measures, both assessing core symptoms of depression and anxiety with similar internal structure in adolescent samples [39]. Standardisation places scores on a common metric, allowing the measures to be combined whilst preserving individual differences to maximise sample size and comparability across samples. To evaluate predictive and incremental validity, we then conducted a set of hierarchical multiple regressions using the combined sample. The three lifestyle domains (Positive Activity, Unhealthy Diet, Substance Use) were entered in Step 1 to assess their unique and combined predictive contributions to symptoms of depression, anxiety, and wellbeing. Negative Social Experiences was added in Step 2 to determine whether it accounted for additional variance in these outcomes beyond lifestyle behaviours, and so to provide evidence of incremental validity. Age, biological sex, and deprivation were added in Step 3 as covariates to examine whether the effects observed in Step 2 remain. Standardised estimates are reported.

Fourth, to examine whether sex (a binary variable) moderated the associations between lifestyle behaviours, negative social experiences, and mental health and wellbeing, we conducted a set of multiple regressions to examine the interaction between each independent variable (Positive Activity, Unhealthy Diet, Substance Use, and Negative Social Experiences; all mean-centred) and sex on levels of depression, anxiety, and wellbeing. Main effects of each independent variable and the interaction term (calculated as the byproduct of each independent variable*sex) were included. Significant interactions were plotted separately for males and females.

Fifth, to examine whether developmental stage (a binary variable) was a significant moderator, we conducted a separate set of multiple regressions as the models for sex, with the interaction term calculated as the byproduct of each independent variable*developmental stage. Significant interactions were plotted separately for the mid-adolescence and late-adolescence samples.

For the interaction models of biological sex and developmental stage, missing data were handled using listwise deletion. Multicollinearity among the predictors and their interaction terms was assessed using variance inflation factors (VIFs); all VIF values were below commonly accepted thresholds (< 5), indicating no multicollinearity concerns.

## Results

### Descriptive statistics

Table 1 presents the means and SDs of PEEL items in descending order for each sample. In general, positive activities were reported as more frequent in both samples, followed by unhealthy diet, negative social experiences, and substance use.

**Table 1:** Means and standard deviations of the PEEL items, ranked from the most to the least frequent in each sample

Late adolescence sample				Mid-adolescence sample			
Items	*n*	Mean	Std. Deviation	Items	*n*	Mean	Std. Deviation
Helping family/friends	378	2.87	1.14	Fun with friends	514	2.63	1.25
Eating ultra-processed foods	377	2.74	1.24	Helping family/friends	516	2.39	1.20
Fun with friends	378	2.59	1.23	Sport or exercise	514	2.27	1.25
Sport or exercise	378	2.21	1.31	Eating ultra-processed foods	516	2.14	1.23
Hobby or creative activities	378	2.21	1.34	Hobby or creative activities	517	2.13	1.35
Feeling less smart than others	377	2.14	1.33	Drinking fizzy drinks	512	1.65	1.27
Reading or writing for fun	378	2.03	1.44	Feeling less smart than others	509	1.45	1.32
Drinking fizzy drinks	376	1.96	1.31	Religious activity	507	1.22	1.50
Religious activity	378	1.56	1.72	Reading or writing for fun	513	1.17	1.31
Volunteer/work outside of home	378	1.41	1.20	Volunteer/work outside of home	515	0.93	1.11
Drinking alcohol	378	1.24	1.08	Trouble with teachers for bad behaviour	516	0.83	1.05
Missing school without parents knowing	377	1.19	1.28	Teased about sexual topics	520	0.63	1.08
Meditation	378	1.13	1.14	Meditation	516	0.61	1.00
Vaping or smoking	377	1.12	1.33	Put down for my ethnicity	515	0.57	1.06
Teased about sexual topics	377	0.80	0.88	Being bullied	511	0.52	0.95
Trouble with teachers for bad behaviour	377	0.75	0.96	Drinking alcohol	510	0.47	0.90
Put down for my ethnicity	377	0.74	0.88	Missing school without parents knowing	520	0.42	0.91
Being bullied	377	0.70	0.83	Vaping or smoking	509	0.32	0.83
Taking illegal drugs	378	0.68	0.81	Taking illegal drugs	508	0.27	0.80

### Validity and reliability of the PEEL

Exploratory factor analyses of the PEEL are presented in Table 2. The initial exploratory factor analysis using data from the late-adolescence sample suggested a four-factor solution of the PEEL (see Supplementary Table 1 for full results). As ‘Volunteer/work outside of home’ and ‘Feeling less smart than others’ had low loadings on all factors, and ‘Religious activity’ was the only item loading on factor four, we removed the three items and reran the factor analysis. The updated factor analysis suggested a three-factor solution: Positive Activity (6 items), Unhealthy Diet (2 items), and Risky Activity (8 items). The revised MAP test suggested a two-factor solution, and the original MAP criterion supported a three-factor solution, with the eigenvalue for the third factor exceeding the Kaiser criterion (1.31). The three-factor model was retained as it captures the conceptual distinctions between the second and third factors.

**Table 2:** Exploratory factor analyses of the PEEL

Items	Late adolescence sample			Mid-adolescence sample			Combined sample (first half)		
	PEEL factors			PEEL factors			PEEL factors		
	Risky A.	Pos. Act.	Diet	Risky A.	Pos. Act.	Diet	Risky A.	Pos. Act.	Diet
Being bullied	0.886	-0.013	-0.096	0.528	-0.053	-0.044	0.740	0.101	0.187
Trouble with teachers for bad behaviour	0.859	-0.036	-0.031	0.440	0.048	0.356	0.640	0.206	0.332
Put down for my ethnicity	0.777	0.107	-0.138	0.577	0.040	-0.111	0.701	0.178	0.020
Taking illegal drugs	0.776	0.078	0.023	0.740	-0.074	-0.092	0.832	0.185	0.179
Teased about sexual topics	0.759	0.039	0.000	0.632	-0.014	0.029	0.695	0.177	0.123
Missing school without parents knowing	0.684	0.035	0.142	0.601	-0.016	0.059	0.736	0.124	0.426
Drinking alcohol	0.555	0.064	0.090	0.751	-0.042	0.131	0.754	0.176	0.317
Vaping or smoking	0.516	-0.050	0.188	0.678	-0.118	0.092	0.709	0.058	0.287
Hobby or creative activities	0.002	0.726	-0.169	0.079	0.709	-0.147	0.214	0.741	-0.038
Sport or exercise	0.046	0.603	-0.004	-0.024	0.502	0.056	0.152	0.766	0.031
Helping family/friends	-0.071	0.646	0.257	-0.111	0.566	-0.010	0.155	0.638	0.318
Fun with friends	-0.037	0.571	0.354	-0.158	0.610	0.179	0.106	0.724	0.311
Meditation	0.298	0.544	-0.169	0.480	0.178	-0.378			
Reading or writing for fun	0.154	0.530	-0.159	0.137	0.357	-0.416			
Eating ultra-processed foods	0.264	0.027	0.511	-0.004	0.347	0.417	0.283	0.239	0.837
Drinking fizzy drinks	0.363	-0.027	0.476	0.211	0.203	0.455	0.373	0.163	0.782

Extraction Method: Principal Axis Factoring. Rotation Method: Direct Oblimin. Loadings < .|40| are greyed out. Items excluded in specific analyses were shaded. Pos. Act. = Positive Activity; Diet = Unhealthy Diet; Risky A. = Risky Activity

The exploratory factor analysis of the remaining 16 items in the mid-adolescence sample suggested that ‘Meditation’ cross-loaded between Risky Activity and Unhealthy Diet (difference in *|*loadings*|* = 0.102), and ‘Reading or writing for fun’ cross-loaded between Positive Activity and Unhealthy Diet (difference in *|*loadings*|* = 0.059). Because these cross-loadings lacked conceptual coherence and the patterns were not consistent across the samples, both items were removed from Positive Activity, leaving four conceptually coherent items. A final exploratory factor analysis of the 14 retained items, using data from both samples, supported the three-factor solution.

After inspecting the loadings, the eight items comprising Risky Activity were grouped into two conceptually distinct categories: Substance Use (3 items) and Negative Social Experiences (5 items). Although these items loaded on a single factor empirically in the EFA, this conceptual restructuring could improve interpretability in subsequent analyses. In addition, differentiating them allowed us to separate modifiable lifestyle behaviour from social-contextual exposures, which reduces potential confounding, enhances conceptual coherence, and supports clearer interpretation of predictive and incremental validity analyses. The two subscales were strongly correlated (*r* = .69, *p* < .001) but represented distinct domains.

Confirmatory factor analysis conducted on the second half of the combined sample showed that the four-factor structure demonstrated an adequate model fit (CFI = 0.940, TLI = 0.917, RMSEA = 0.064). The resulting four subscales, Positive Activity (4 items), Unhealthy Diet (2 items), Substance Use (3 items), and Negative Social Experiences (5 items), demonstrated good internal consistency (Cronbach’s alpha = 0.71, 0.60, 0.80 and 0.79) and test-retest reliability (intraclass correlation with two-way mixed effects and absolute agreement = 0.86, 0.89, 0.90 and 0.88), based on common thresholds. As there were only two items loaded on Unhealthy Diet with a Cronbach’s alpha of just 0.60, this could be used as an index of unhealthy diet rather than a comprehensive subscale. The two items were moderately correlated with each other (*r* = .42, *p* < .001). There are significant sex and developmental stage differences in Unhealthy Diet, Substance Use, and Negative Social Experiences (all *p* < .001; see Table 3).

**Table 3:** Sex and developmental stage differences in PEEL subscales

	Male			Female			
	*N*	Mean	SD	*N*	Mean	SD	Diff. in mean
Positive activity	346	2.41	0.89	516	2.42	0.94	0.01
Unhealthy diet	349	1.94	0.98	517	2.19	1.13	0.25*
Substance use	348	0.39	0.72	511	0.77	0.90	0.37*
Negative social experiences	346	0.54	0.72	512	0.75	0.74	0.21*

	**Mid-adolescence**			**Late adolescence**			
	N	Mean	SD	N	Mean	SD	Diff. in mean
Positive activity	501	2.38	0.91	378	2.47	0.94	0.09
Unhealthy diet	509	1.89	1.02	376	2.35	1.1	0.46*
Substance use	501	0.33	0.69	377	1.01	0.9	0.68*
Negative social experiences	501	0.56	0.66	377	0.83	0.82	0.27*

* Indicates *p* < .001 in independent t-tests. Reference group is male/mid-adolescence sample (i.e., Differences in mean = female – male/late adolescence sample – mid-adolescence sample)

### Associations between positive and risky lifestyle behaviours, negative social experiences and mental health and wellbeing

Results from the hierarchical multiple regressions are presented in Table 4.

For depression, all three lifestyle factors were significant predictors in Step 1: Positive Activity was negatively (β = − 0.20, *p* < .001), and Unhealthy Diet (β = 0.22, *p* < .001) and Substance Use (β = 0.18, *p* < .001) were positively associated with depressive symptoms. When Negative Social Experiences was added in Step 2, it most strongly predicted higher levels of depression (β = 0.35, *p* < .001). The first two lifestyle factors remained significant (β = − 0.21 and 0.16, both *p* < .001), whereas Substance Use became non-significant (*p* = .462). The patterns of associations remained the same after adjusting for age, sex, and deprivation in Step 3.

For anxiety, all three lifestyle factors were also significant predictors in Step 1 (β = − 0.11, 0.18, and 0.19, respectively, all *p* < .001). In Step 2, Negative Social Experiences most strongly predicted higher levels of anxiety (β = 0.35, *p* < .001), and Positive Activity and Unhealthy Diet remained significant (β = − 0.13 and 0.13, both *p* < .001). Substance Use was no longer significant (*p* = .459). The patterns of associations remained the same after adjusting for age, sex, and deprivation in Step 3.

For wellbeing, Positive Activity was the strongest predictor in Step 1 (β = 0.53, *p* < .001). Unhealthy Diet (β = − 0.07, *p* = .28) and Substance Use (β = − 0.12, *p* < .001) were both negatively associated with wellbeing. In Step 2, Negative Social Experiences was significantly associated with lower wellbeing (β = − 0.21, *p* < .001), and Positive Activity remained the strongest predictor (β = 0.54, *p* < .001). Unhealthy Diet (*p* = .312) and Substance Use (*p* = .846) were no longer significantly associated with wellbeing. The patterns of associations remained the same after adjusting for age, sex, and deprivation in Step 3.

In addition, we conducted sensitivity analyses in which Substance Use and Negative Social Experiences were combined into a single Risky Activity factor, as identified from the EFA. Findings showed consistent patterns of association with comparable effect sizes (see Supplementary Table 2 for full results). We also conducted another set of sensitivity analyses comparing the patterns of associations in participants who completed the RCADS-25 with those who completed the DASS-21; again, the results demonstrated similar patterns of association with comparable effect sizes (see Supplementary Table 3 for full results).

**Table 4:** Associations between lifestyle factors, Negative social experiences, and symptoms of depression, anxiety, and wellbeing

	Depression						Anxiety						Wellbeing					
	Step 1		Step 2		Step 3		Step 1		Step 2		Step 3		Step 1		Step 2		Step 3	
	β	*p*-value	β	*p*-value	β	*p*-value	β	*p*-value	β	*p*-value	β	*p*-value	β	*p*-value	β	*p*-value	β	*p*-value
Positive Activity	**− 0.20**	< 0.001	**− 0.21**	< 0.001	**− 0.23**	< 0.001	**− 0.11**	< 0.001	**− 0.13**	< 0.001	**− 0.13**	< 0.001	**0.53**	< 0.001	**0.54**	< 0.001	**0.56**	< 0.001
Unhealthy Diet	**0.22**	< 0.001	**0.16**	< 0.001	**0.20**	< 0.001	**0.18**	< 0.001	**0.13**	< 0.001	**0.18**	< 0.001	**− 0.07**	0.028	− 0.03	0.312	− 0.05	0.162
Substance Use	**0.18**	< 0.001	− 0.03	0.462	0.08	0.136	**0.19**	< 0.001	− 0.03	0.459	0.03	0.596	**− 0.12**	< 0.001	0.01	0.846	0.00	0.958
Negative social experiences			**0.35**	< 0.001	**0.29**	< 0.001			**0.35**	< 0.001	**0.28**	< 0.001			**− 0.21**	< 0.001	**− 0.19**	< 0.001
Age					**− 0.12**	0.003					**− 0.09**	0.028					0.02	0.544
Biological sex					− 0.05	0.151					**− 0.08**	0.042					0.00	0.987
Deprivation					0.02	0.650					0.01	0.694					0.02	0.544
	R^2^		ΔR² from S1		ΔR² from S2		R^2^		ΔR² from S1		ΔR² from S2		R^2^		ΔR² from S1		ΔR² from S2	
	0.113, *p* < .001		0.062, *p* < .001		0.017, *p* = .002		0.084, *p* < .001		0.062, *p* < .001		0.016, *p* = .006		0.262, *p* < .001		0.022, *p* < .001		0.001, *p* = .872	

Positive Activity, Unhealthy Diet, and Substance Use were added in Step 1; Negative Social Experiences was added in Step 2; age, sex, and deprivation were added in Step 3. β = standardised coefficient beta. Significant coefficients are in bold

### The moderating effect of biological sex

**Fig. 1:**
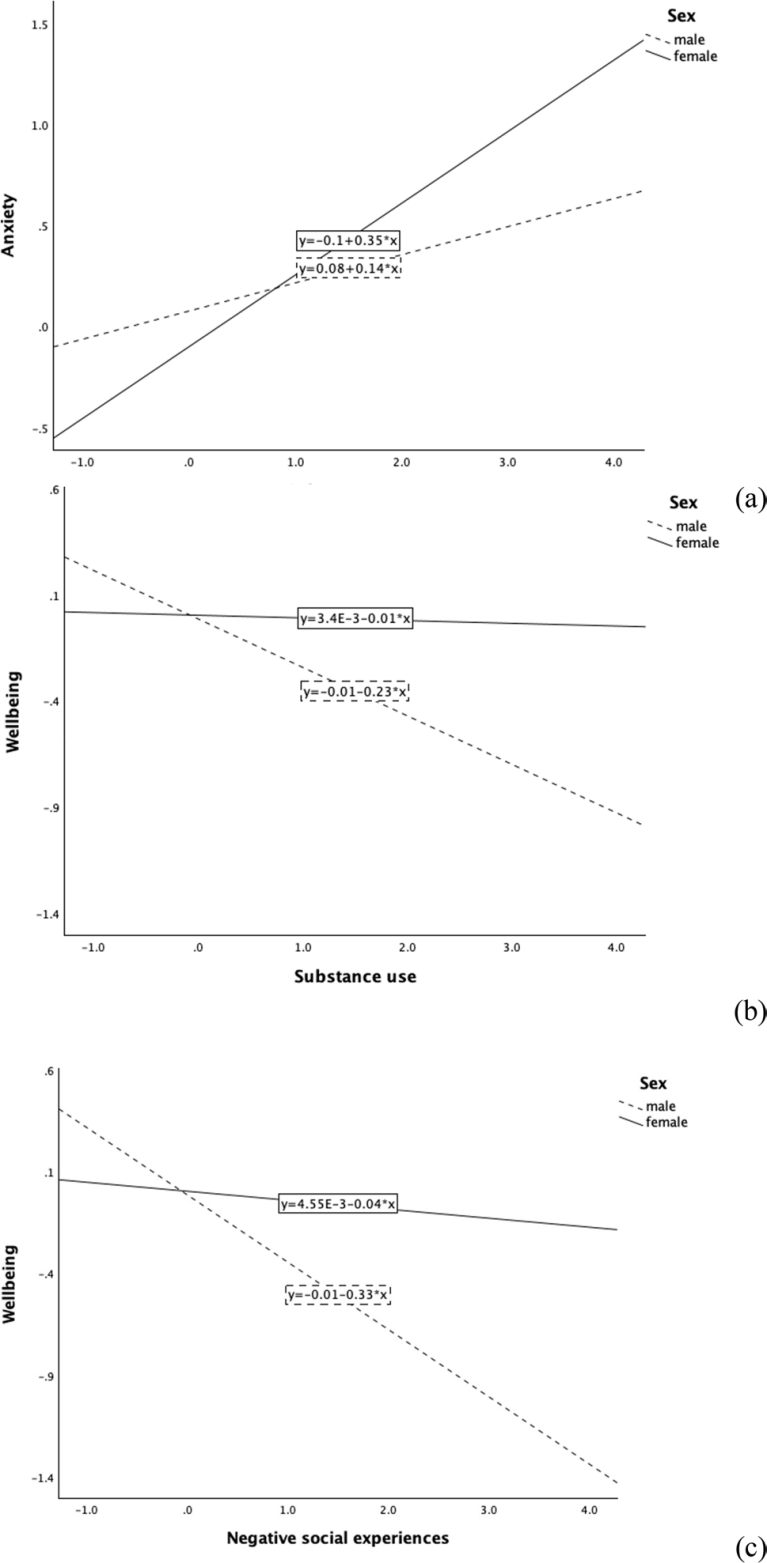
Interaction plots showing sex differences in the associations between (**a**) Substance Use and anxiety symptoms, (**b**) Substance Use and wellbeing, and (**c**) Negative Social Experiences and wellbeing. The x-axis shows mean-centred predictor variables, with separate lines plotted for male and female. Outcome values represent observed data; the values are not adjusted for covariates in these plots

Interaction analyses suggested that sex significantly moderated the association between Substance Use and symptoms of anxiety (*p* = .013; Table 5), where the positive association was stronger in females than in males (Fig. 1a). Sex also significantly moderated the association between Substance Use (*p* = .020), as well as Negative Social Experiences (*p* = .003), and wellbeing, where the negative associations were stronger in males than in females (Fig. 1b and c). However, biological sex was not a significant moderator of the associations between any of the positive and risky lifestyle factors or Negative Social Experiences and symptoms of depression.

**Table 5:** Significant interactions between lifestyle factors, negative social experiences, and *biological sex* on anxiety symptoms and wellbeing

Anxiety	β	*p*-value	*R* ^2^
			0.063, *p* < .001
Substance Use	0.12	0.055	
Sex	**− 0.09**	0.012	
Substance Use * sex	**0.15**	0.013	
**Wellbeing**			0.011, *p* = .031
Substance Use	**− 0.19**	0.004	
Sex	0.01	0.813	
Substance Use * sex	**0.15**	0.020	
**Wellbeing**			0.019, *p* < .001
Negative Social Experiences	**− 0.24**	< 0.001	
Sex	0.01	0.787	
Negative Social Experiences * sex	**0.16**	0.003	

β = standardised coefficient beta. Significant coefficients are in bold

### The moderating effect of developmental stage

**Fig. 2:**
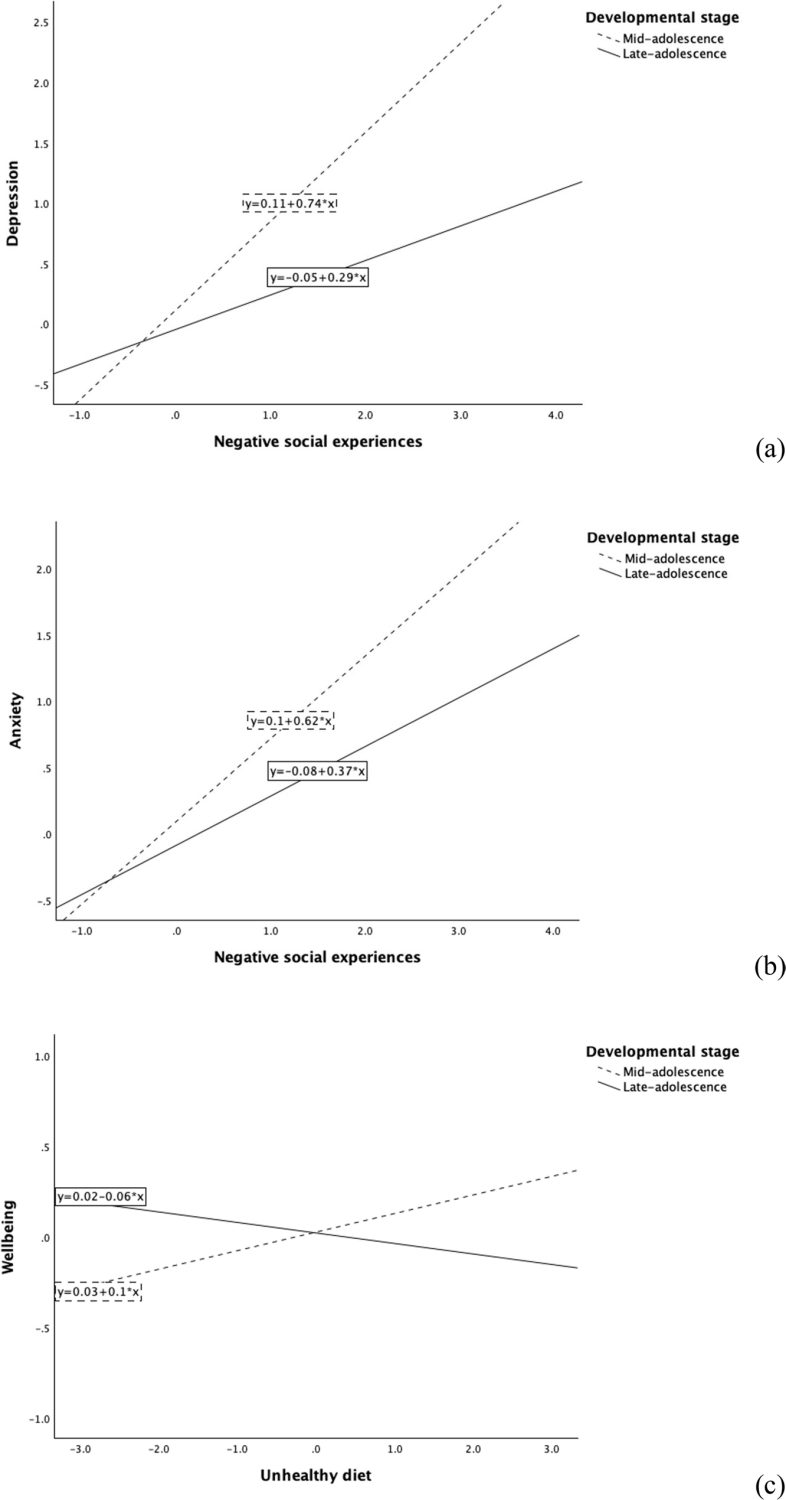
Interaction plots showing developmental stage differences in the associations between (**a**) Negative Social Experiences and depressive symptoms, (**b**) Negative Social Experiences and anxiety symptoms, and (**c**) Unhealthy Diet and wellbeing. The x-axis shows mean-centred predictor variables, with separate lines plotted for the mid- and late-adolescence samples. Outcome values represent observed data; the values are not adjusted for covariates in these plots

Developmental stage was a significant moderator of the associations between Negative Social Experiences with symptoms of both depression (*p* < .001) and anxiety (*p* = .004; Table 6), where the negative associations with depressive and anxiety symptoms were stronger in younger (i.e., mid-adolescence) than older adolescents (i.e., mid-adolescence; Fig. 2a and b). For wellbeing, there was a significant interaction between developmental stage and Unhealthy Diet (*p* = .003), where it was positively related to wellbeing in younger adolescents but negatively in older adolescents (Fig. 2c).

**Table 6:** Significant interactions between positive and risky lifestyle factors, Negative social experiences, and *age* on symptoms of depression, anxiety, and wellbeing

Depression	β	*p*-value	*R* ^2^
			0.146, *p* < .001
Negative Social Experiences	**0.54**	< 0.001	
Developmental stage	**− 0.08**	0.014	
Negative Social Experiences * developmental stage	**− 0.25**	< 0.001	
**Anxiety**			0.134, *p* < .001
Negative Social Experiences	**0.40**	< 0.001	
Developmental stage	**− 0.09**	0.006	
Negative Social Experiences * developmental stage	**− 0.10**	0.004	
**Wellbeing**			0.007, *p* = .026
Unhealthy Diet	0.03	0.408	
Developmental stage	0.03	0.432	
Unhealthy Diet * developmental stage	**− 0.11**	0.003	

β = standardised coefficient beta. Significant coefficients are in bold

## Discussion

In the present study, we developed and examined the psychometric properties of the Personal Experiences in Everyday Life (PEEL) questionnaire, a new self-report tool designed to address limitations in existing assessments of adolescent lifestyle behaviours and important contextual exposures. The PEEL provides a single measure capturing positive and risky lifestyle behaviours and key personal-social experiences that are most salient to adolescent mental health. It is a practical measurement tool that reduces participant burden and supports consistent assessment across studies. We also tested the PEEL’s validity in predicting variations in symptoms of depression, anxiety, and psychological wellbeing in youth aged 13–25. The analyses revealed five important findings.

First, the exploratory factor analysis resulted in a psychometrically sound measure consisting of 14 items. Nine lifestyle behaviour items loaded on three factors: Positive Activity (4 items), Unhealthy Diet (2 items), Substance Use (3 items), and five items loaded onto the fourth factor, capturing Negative Social Experiences. The PEEL was completed by both younger and older adolescents from diverse ethnic backgrounds. The factor structure was consistent across these age groups, indicating that the PEEL performs consistently and is psychometrically valid across a wide age range and diverse ethnic groups and, thus, is suitable for use in heterogeneous adolescent populations.

Second, Positive Activity showed a small but consistent negative association with depressive and anxiety symptoms, and a substantially stronger positive association with wellbeing. This factor captured adolescents’ engagement in creative/hobby activities, sport or exercise, helping family or friends, and spending time with friends. These activities span physical, social, and mastery-oriented domains, all of which have been proposed to support positive psychological functioning. The weak negative associations with mental health symptoms, alongside a strong positive association with wellbeing observed in the present study, align with evidence suggesting that these activities may promote adolescents’ positive psychological adjustment more reliably than they reduce depression and anxiety. For example, research shows that physical activity is associated with improvements in self-esteem [40], emotion regulation [41], and cognitive control [42], while engagement in hobbies and creative activities provides opportunities for autonomy and competence development, which are central to wellbeing [43]. Helping behaviours can support self-worth, social connectedness, and a sense of purpose [24], and positive peer interactions are consistently linked to higher life satisfaction and emotional wellbeing [44]. Taken together, these findings suggest that regular participation in enjoyable, social, and mastery-building activities may play a particularly important role in supporting adolescents’ general wellbeing, even if their impact on symptoms of anxiety and depression is relatively modest. Consequently, these activities and experiences may represent more effective targets for promoting flourishing than preventing mental ill-health.

Third, Unhealthy Diet factor showed weak but consistent positive associations with depressive and anxiety symptoms, which remained significant after accounting for Negative Social Experiences. Adolescents in this study reported consuming sugary drinks and ultra-processed foods at least a few times a week, indicating that these behaviours were frequent. These findings align with evidence linking unhealthy dietary patterns to poorer mental health outcomes in adolescents [45], potentially reflecting indirect effects on metabolic health and the role of gut-brain-microbiome pathways that are increasingly hypothesised in supporting brain function and modulating psychiatric risks [46, 47]. For wellbeing, however, the association differed by developmental stage. Unhealthy dietary habits were associated with higher wellbeing in mid-adolescence, but with lower wellbeing in late adolescence and early adulthood.

This crossover interaction should be interpreted cautiously as an exploratory finding, suggesting that the meaning and context of unhealthy eating may vary across developmental stages. In mid-adolescence, consuming sugary drinks and ultra-processed foods may function as a social or enjoyable activity [48]. In contrast, older adolescents may be more aware of the health implications of these dietary choices. As a results, they may lean towards healthier diet choices [49]. When they do eat unhealthily, this increased awareness could result in feelings of guilt or conflict with personal goals and may help explain the shift toward a link with lower wellbeing later in youth. Alternatively, age-related differences in reporting, social norms, and health-related expectations, as well as other unmeasured confounders, may also influence how dietary behaviours relate to wellbeing over time.

In addition, given that the Unhealthy Diet domain consists of only two items and showed modest internal consistency (α = 0.60), these associations, particularly the developmental stage and wellbeing interaction, should be interpreted with additional caution. The limited item coverage means this domain functions more as an index than a comprehensive subscale, which may reduce measurement precision and increase measurement-related variability when comparing effects across these two developmental stages.

Fourth, in the initial model, Substance Use was weakly associated with higher depressive and anxiety symptoms and lower wellbeing. However, these associations varied by sex. For females, substance use was more strongly associated with higher anxiety symptoms. For males, substance use was more strongly associated with lower wellbeing. These findings suggest that substance use may have different psychological functions depending on sex. For example, females may be more likely to use substances as a maladaptive emotional-regulation strategy [50, 51]. For males, substance use is more often linked to sensation seeking and experimentation or peer-status [52, 53], which can impact positive routines (e.g., school), and reduce engagement in positive activities (e.g., exercise, hobbies), and, in turn, reduce wellbeing.

Importantly, when Negative Social experiences, including bullying, teacher conflict, ethnicity-related teasing, sexual teasing, and truancy, were added to the model, the associations between substance use and mental health and wellbeing were no longer significant. This indicates that the apparent link between substance use and mental health is largely explained by shared social-interpersonal risk. Young people who use substances more frequently also report higher levels of bullying, school conflict, and general social adversity [54]. These findings indicate that substance use may function more as a marker of psychosocial strain rather than an independent risk factor for mental health symptoms, once social experiences are accounted for [55]. Taken together, negative social experiences emerge as a key driver of associations with mental health, highlighting the vital role of social context alongside lifestyle behaviours in understanding youth mental health.

Fifth, Negative Social Experiences emerged as the strongest predictors of depressive and anxiety symptoms and reduced wellbeing in this study, even after accounting for lifestyle behaviours. This finding demonstrates clear incremental validity of this domain within the PEEL and is consistent with evidence that interpersonal and social adversity is a key driver of youth mental health [56]. These associations were also stronger in mid-adolescence, indicating increased sensitivity to such negative experiences at a younger age. Overall, these findings highlight the crucial role of social and interpersonal context in adolescent mental health and the particular vulnerability of younger adolescents to these experiences. However, these findings should be interpreted in light of the analytical decisions underpinning the PEEL’s factor structure. Although the exploratory factor analysis indicated that Substance Use and Negative Social Experiences loaded onto a single *Risky Activity* factor, we separated these domains based on their conceptual distinction and relevance for youth mental health. The strong correlation between these domains (*r* = .69) suggest substantial overlap but separating them improves interpretability. It also allows examination of whether behavioural risks (e.g., substance use) and social-contextual risks (e.g., bullying, teacher conflict) show unique associations with mental health outcomes. While this approach may limit discriminant validity, the observed differential associations support the value of this distinction. Future research may help clarify whether these domains represent separate constructs or form a single higher-order risk factor.

A key strength of this study is the inclusion of two independent samples spanning mid- to late-adolescence and early adulthood - a developmental period during which lifestyle behaviours become established and set foundations for future mental (and physical) health. The samples were ethnically diverse, improving generalisability and better reflecting the broad contexts in which lifestyle determinants of mental health operate. The factor structure replicated across both age groups, indicating that the PEEL is a psychometrically robust measure across nearly the full adolescent age range (approximately 10–24 years). In addition, by assessing lifestyle behaviours and negative social experiences within a single brief instrument, the PEEL provides a comprehensive yet practical tool for research on lifestyle determinants of mental health, reducing participant burden while allowing assessment of multiple relevant domains.

Despite these strengths, several limitations should be noted. First, the analyses were cross-sectional, preventing conclusions about the direction or temporal order of associations between lifestyle behaviours, social experiences, and mental health and wellbeing. Therefore, longitudinal designs will be needed to establish how these relationships develop over time and to identify the mechanisms through which lifestyle factors affect adolescent mental health. Second, all measures were self-reported at the same time point, raising the possibility of common method variance. Selection bias is another possibility, as the late-adolescent sample was recruited through schools and university pools, which may under-represent marginalised youth. Additionally, some demographic measures in this study are context-specific (e.g., free school meals) and will require adaptations to contexts outside the UK. Although the samples were ethnically diverse, data were collected solely in the UK, and some demographic indicators, such as free school meals and school‑related items, are context‑specific, which could reduce the generalisability of this research. Future cross‑cultural validation will be important to determine whether the PEEL’s structure and correlates apply in non‑UK contexts.

While combining lifestyle behaviours and negative social experiences in the PEEL broadens the assessment, it also means that the PEEL is not a pure “lifestyle” instrument, representing both a strength and a conceptual limitation. Third, although substance use and negative social experiences were conceptualised as distinct domains in this study, the factor analysis did not separate them into independent constructs. This may indicate that these behaviours and experiences tap into the same dimension during adolescence, rather than reflecting multiple underlying constructs.

A further limitation is that depressive and anxiety symptoms were assessed using two different instruments (RCADS-25 and DASS-21), which we then combined to maximise sample size. This may limit clinical comparability across samples, as the measures differ in their item content, particularly for anxiety. While the RCADS-25 and DASS-21 depression subscales share overlapping item content reflecting core depressive features (e.g., low mood and anhedonia), anxiety-related item content is more unique to each of the two measures. The RCADS-25 draws on DSM-defined anxiety disorders’ symptoms (e.g., excessive worry, social and situational fears, and panic-related symptoms), whereas the DASS-21 anxiety subscale primarily captures physiological arousal (e.g., trembling, mouth dryness) and feelings of imminent panic. Therefore, pooled anxiety scores should be interpreted as reflecting general anxiety symptoms severity rather than specific anxiety disorder phenotypes or symptom dimensions. Despite this, sensitivity analyses separating the RCADS and DASS samples identified similar patterns of associations, suggesting that the results were not driven by instrument choice, supporting the stability and reliability of the findings.

Finally, we acknowledge that conducting multiple regression and interaction analyses may increase the risk of Type I error. Nevertheless, we focus our interpretation on the overall pattern of results and effect sizes, which highlight the potential impact of specific behaviours and experiences.

Overall, the PEEL provides a practical and efficient tool for assessing multiple lifestyle behaviours and negative social experiences in a single brief measure, making it well-suited for research, especially in extensive adolescent assessment batteries. By providing a quick profile of adolescents’ daily activities and experiences, the PEEL will be useful in studies investigating multiple concurrent behaviours and their associations with mental health. The findings also highlight several domains that warrant further investigation in future longitudinal studies, such as positive activities, substance use, and dietary choices. Given the particularly strong links between negative personal and social experiences and mental health, especially in younger adolescents, these experiences should be routinely considered in both youth mental health research and practice. The empirical clustering of Substance Use and Negative Social Experiences into a single factor points to a broader underlying dimension of risky and adverse experiences in adolescence. Future research could build on this insight by examining higher-order or bifactor models to further refine the structure of adolescent risk and adversity.

## Conclusions

This study shows that the PEEL is a psychometrically valid and practical tool for capturing adolescents’ lifestyle behaviours and negative social experiences across a wide developmental range. Positive Activity was the strongest correlate of wellbeing, while negative social experiences were most strongly and robustly associated with depressive and anxiety symptoms. Unhealthy Diet was also weakly but independently linked to these symptoms. In contrast, the associations between substance use and mental health were fully explained by co-occurring social and interpersonal difficulties, highlighting the importance of considering lifestyle habits within their broader context.

## Supplementary Information

.Supplementary Material 1.

## Data Availability

The datasets generated and/or analysed during the current study will be deposited in a public archive within 2 years after the end of the project (current project end date: 31 Mar 2026). Currently, the datasets used in this study are available from the corresponding author on reasonable request.

## Abbreviations

CFA: Confirmatory Factor Analysis
CFI: Comparative Fit Index
DASS-21: the Depression, Anxiety, and Stress Scale
EFA: Exploratory Factor Analysis
MAP: the Minimum Average Partial test
PEEL: the Personal Experiences in Everyday Life (PEEL) questionnaire
RCADS-25: the Revised Child Anxiety and Depression Scale 25 – Youth Version
RMSEA: Root Mean Square Error of Approximation
SD: Standard Deviation
TLI: Tucker-Lewis Index
VIF: variance inflation factor
WEMWBS: the Warwick-Edinburgh Mental Wellbeing Scale
