# Supplementary material for: Assessing key mental health-related lifestyle behaviours and experiences in a single scale: a validation of the Personal Experiences in Everyday Life (PEEL) questionnaire
Source: BMC Pediatr. 2026 Mar 30;26:421. doi: 10.1186/s12887-026-06790-x (PMC13154467; doi:10.1186/s12887-026-06790-x)
Supplement: Supplementary file 1 — .Supplementary Material 1. [file 12887_2026_6790_MOESM1_ESM.docx]

**Supplementary materials**

**Title: Assessing key mental health-related lifestyle behaviours and experiences in a single scale: A validation of the *Personal Experiences in Everyday Life* (PEEL) questionnaire.**

Supplementary Table 1. Initial exploratory factor analyses of the PEEL.

|  | **Late adolescence sample** | | | |
| --- | --- | --- | --- | --- |
| **Items** | Initial factor analysis | | | |
|  | 1 | 2 | 3 | 4 |
| Being bullied | 0.853 | -0.022 | 0.042 | 0.089 |
| Trouble with teachers for bad behaviour | 0.813 | -0.040 | 0.094 | 0.080 |
| Put down for my ethnicity | 0.772 | 0.085 | -0.016 | 0.152 |
| Taking illegal drugs | 0.734 | 0.139 | 0.032 | -0.182 |
| Teased about sexual topics | 0.715 | 0.059 | 0.090 | 0.007 |
| Missing school without parents knowing | 0.603 | 0.014 | 0.297 | 0.057 |
| Drinking alcohol | 0.519 | 0.165 | 0.007 | -0.407 |
| Vaping or smoking | 0.459 | 0.049 | 0.126 | -0.438 |
| Hobby or creative activities | 0.045 | 0.683 | -0.158 | 0.042 |
| Sport or exercise | 0.049 | 0.649 | -0.074 | -0.121 |
| Helping family/friends | -0.130 | 0.631 | 0.292 | 0.110 |
| Fun with friends | -0.121 | 0.621 | 0.312 | -0.143 |
| Meditation | 0.330 | 0.545 | -0.152 | 0.097 |
| Reading or writing for fun | 0.183 | 0.483 | -0.095 | 0.193 |
| Volunteer/work outside of home* | 0.287 | 0.356 | 0.022 | 0.123 |
| Eating ultra-processed foods | 0.102 | 0.020 | 0.654 | 0.071 |
| Drinking fizzy drinks | 0.230 | -0.005 | 0.519 | -0.035 |
| Feeling less smart than others* | 0.325 | -0.044 | 0.351 | -0.106 |
| Religious activity* | 0.285 | 0.206 | 0.092 | 0.526 |

Note. Extraction Method: Principal Axis Factoring. Rotation Method: Direct Oblimin. Loadings < .|40| are greyed out. Items excluded from subsequent analyses are denoted with an asterisk (*)**.**

Supplementary Table 2. Sensitivity analyses showing the associations between lifestyle factors (with a single Risky Activity factor), Negative social experiences, and symptoms of depression, anxiety, and wellbeing.

|  | **Depression** | | | | **Anxiety** | | | | **Wellbeing** | | | |
| --- | --- | --- | --- | --- | --- | --- | --- | --- | --- | --- | --- | --- |
|  | **Step 1** | | **Step 2** | | **Step 1** | | **Step 2** | | **Step 1** | | **Step 2** | |
|  | β | *p*-value | β | *p*-value | β | *p*-value | β | *p*-value | β | *p*-value | β | *p*-value |
| Positive Activity | **-.23** | <.001 | **-.23** | <.001 | **-.13** | <.001 | **-.13** | <.001 | **.55** | <.001 | **.55** | <.001 |
| Unhealthy Diet | **.20** | <.001 | **.21** | <.001 | **.18** | <.001 | **.19** | <.001 | -.05 | .135 | -.06 | .110 |
| Risky Activity | **.28** | <.001 | **.34** | <.001 | **.24** | <.001 | **.30** | <.001 | **-.16** | <.001 | **-.18** | <.001 |
| Age |  | | **-.14** | <.001 |  |  | **-.12** | .004 |  |  | .04 | .247 |
| Biological sex |  | | -.05 | .149 |  |  | **-.08** | .040 |  |  | .00 | .974 |
| Deprivation |  | | .02 | .538 |  |  | .02 | .596 |  |  | .01 | .664 |
|  | R^2^ | | ΔR² | | R^2^ | | ΔR² | | R^2^ | | ΔR² | |
|  | .164, *p* < .001 | | .023, *p* < .001 | | .116, *p* < .001 | | .023, *p* < .001 | | .290, *p* < .001 | | .002, *p* = .671 | |

Note. Substance Use and Negative Social Experiences were combined into a single Risky Activity as identified in the EFA.

Positive Activity, Unhealthy Diet, and Risky Activity were added in Step 1; age, sex, and deprivation were added in Step 2. β = standardised coefficient beta. Significant coefficients are in bold.

Supplementary Table 3. Sensitivity analyses showing the associations between lifestyle factors, Negative social experiences, and symptoms of depression and anxiety, separately for participants who completed the RCADS-25 and the DASS-21.

|  | **Depression** | | | | | | **Anxiety** | | | | | |
| --- | --- | --- | --- | --- | --- | --- | --- | --- | --- | --- | --- | --- |
|  | **Step 1** | | **Step 2** | | **Step 3** | | **Step 1** | | **Step 2** | | **Step 3** | |
|  | β | *p*-value | β | *p*-value | β | *p*-value | β | *p*-value | β | *p*-value | β | *p*-value |
| **RCADS-25 sample (*n* = 743)** | | | | | | | | | | | | |
| Positive Activity | **-.16** | <.001 | **-.18** | <.001 | **-.17** | <.001 | -.06 | .150 | -.08 | .060 | -.07 | .093 |
| Unhealthy Diet | **.29** | <.001 | **.24** | <.001 | **.23** | <.001 | **.23** | <.001 | **.18** | <.001 | **.18** | <.001 |
| Substance Use | **.23** | <.001 | .04 | .360 | .03 | .551 | **.21** | <.001 | -.04 | .347 | .03 | .495 |
| Negative social experiences |  |  | **.36** | <.001 | **.38** | <.001 |  |  | **.31** | <.001 | **.34** | <.001 |
| Age |  | |  | | .07 | .104 |  | |  | | .07 | .090 |
| Biological sex |  | |  | | **-.09** | .021 |  | |  | | **-.13** | .002 |
| Deprivation |  | |  | | -.02 | .680 |  | |  | | -.02 | .530 |
|  | R^2^ | | ΔR² from S1 | | ΔR² from S2 | | R^2^ | | ΔR² from S1 | | ΔR² from S2 | |
|  | .163, *p* < .001 | | .085, *p* < .001 | | .009, *p* = .081 | | .109, *p* < .001 | | .065, *p* < .001 | | .017, *p* = .010 | |
| **DASS-21 sample (*n* = 192)** | | | | | | | | | | | | |
| Positive Activity | **-.27** | <.001 | **-.24** | .001 | **-.21** | .006 | -.13 | .089 | -.08 | .288 | -.03 | .654 |
| Unhealthy Diet | **.15** | .043 | .12 | .090 | .11 | .136 | **.28** | <.001 | **.25** | <.001 | **.22** | .002 |
| Substance Use | **.25** | <.001 | **.17** | .032 | **.16** | .049 | **.15** | .046 | .03 | .689 | .01 | .868 |
| Negative social experiences |  |  | **.22** | .006 | **.24** | .003 |  |  | **.32** | <.001 | **.35** | <.001 |
| Age |  | |  | | .04 | .564 |  | |  | | .09 | .210 |
| Biological sex |  | |  | | .13 | .078 |  | |  | | **.18** | .012 |
| Deprivation |  | |  | | .07 | .350 |  | |  | | .10 | .144 |
|  | R^2^ | | ΔR² from S1 | | ΔR² from S2 | | R^2^ | | ΔR² from S1 | | ΔR² from S2 | |
|  | .162, *p* < .001 | | .039, *p* = .006 | | .023, *p* = .209 | | .129, *p* < .001 | | .082, *p* < .001 | | .052, *p* = .014 | |

Note. Positive Activity, Unhealthy Diet, and Substance Use were added in Step 1; Negative Social Experiences was added in Step 2; age, sex, and deprivation were added in Step 3. β = standardised coefficient beta. Significant coefficients are in bold.
